# Maternal Vaccination as an Essential Component of Life-Course Immunization and Its Contribution to Preventive Neonatology

**DOI:** 10.3390/ijerph15050847

**Published:** 2018-04-25

**Authors:** Naomi Bergin, Janice Murtagh, Roy K. Philip

**Affiliations:** 1 Division of Neonatology, Department of Paediatrics, University Maternity Hospital Limerick (UMHL), Limerick V94 C566, Ireland; bergin.naomi@gmail.com; 2 MSD Ireland Ltd., South County Business Park, Leopardstown, Dublin D18 X5K7, Ireland; janice.murtagh@merck.com

**Keywords:** maternal immunization, life-course immunization, life-long immunization, preventive neonatology, neonatal intensive care, antimicrobial resistance, vaccines, breastfeeding, pregnancy, immunity

## Abstract

Maternal immunisation schedules are increasingly coming under the spotlight as part of the development of lifetime immunisation programmes for the role that they play in improving maternal, foetal, and neonatal health. Maternally-acquired antibodies are critical in protecting infants during the first months of their lives. Maternal immunisation was previously overlooked owing to concerns regarding vaccinations in this untested and high-risk population but is now acknowledged for its potential impact on the outcomes in many domains of foetal and neonatal health, aside from its maternal benefits. This article highlights the role that maternal immunisation may play in reducing infections in preterm and term infants. It explores the barriers to antenatal vaccinations and the optimisation of the immunisation uptake. This review also probes the part that maternal immunisation may hold in the reduction of perinatal antimicrobial resistance and the prevention of non-infectious diseases. Both healthcare providers and expectant mothers should continue to be educated on the importance and safety of the appropriate immunizations during pregnancy. Maternal vaccination merits its deserved priority in a life-course immunization approach and it is perhaps the only immunization whereby two generations benefit directly from a single input. We outline the current recommendations for antenatal vaccinations and highlight the potential advances in the field contributing to “*preventive neonatology*”.

## 1. Introduction

Immunization has been effective in preventing and reducing the number of devastating infections globally. Immunization programmes have traditionally focused on the prevention of childhood illnesses with vaccinations given during infancy and childhood. Infectious diseases continue to be one of the leading causes of morbidity and mortality in the antenatal, postpartum, and neonatal periods. The evidence has demonstrated a higher incidence of infections [1] and a more severe illness course within these groups compared to others. Maternally-acquired antibodies are critical in protecting infants during the first months of their lives. Consequently, the immunization of pregnant women is an important strategy, not only to protect the mothers from infection but also to provide immunity to the young infants [2]. 

Neonates are at increased risk of infections for many reasons but primarily due to their immature immune system and therefore, their relative inability to mount a humoral immune response. This paucity or relative inability argues against the provision of neonatal vaccinations in preventing infections during early infancy and requires a more comprehensive preventative strategy to reduce the morbidity associated with infectious diseases in this vulnerable cohort.

Recent evidence supports the role of a lifetime immunisation schedule [3] and, following from this, there has been an increasing body of work on maternal vaccination schedules. New outbreaks of the influenza pandemic and the resurgence of pertussis have resulted in policy changes and shifts in the health authority recommendations towards vaccines aimed at protecting both pregnant women and their infants in the first months of their lives [4]. A number of additional maternal vaccines that have the potential to combat neonatal infections are also in the pipeline [5]. As the evidence accumulates for the effectiveness of these vaccines in the coming years, there may be an increased acceptance and an increased uptake of maternal vaccinations aimed at reducing neonatal infections. The advent of widespread maternal immunisation will, in turn, lead to the development of the concept of “preventive neonatology”. Maternal vaccination programmes will hopefully undergo a paradigm shift as society and healthcare providers accept and recognize the benefits that are achievable from this strategy, not only for the pregnant woman but also for the developing foetus and the young infant [6]. This review outlines the rationale, evidence, challenges, and perceptions of vaccination during the pregnancy, as well as the recent developments, and will highlight the impact on the current and future clinical practices. 

## 2. Objectives

This narrative review on maternal (antenatal) vaccination aims to discuss the types of vaccines that are available and are approved for the safe general uptake during pregnancy, the vaccines that are recommended during pregnancy based on risk factors and special circumstances, the vaccines that are currently under research and are in development for licensure for maternal-foetal immunization, and the barriers to maternal immunization. In addition, we attempt to review the challenges that maternal vaccinations are facing, posed by the general vaccine hesitancy and the anti-vaccine movements, as well as the need to adapt to modern communication platforms. The unconventional benefits of vaccination during pregnancy, such as the reduction of perinatal antimicrobial resistance and the potential future value in reducing even the non-infectious pathologies during infancy are discussed.

Medline/PubMed, CINAHL, and Web of Science were searched with “English language” as a filter and the search terms were as follows: “maternal immunization” or “maternal vaccination”; “immunization during pregnancy” or “vaccination during pregnancy”; “antenatal immunization” or “antenatal vaccination”; or “immunization”; or “vaccination” and “pregnancy”. The publication limits were set between the years 2000–2018 for human research only. Single case reports were not included, however, other categories such as observational studies, comparative studies, consensus statements from learned societies/governmental agencies, review articles, randomized trials, systematic reviews, and meta-analyses were included. We did not undertake a systematic review or a meta-analysis. The general literature search was conducted by all the authors independently and the corresponding author organised the structured search with pre-agreed criteria/MESH headings.

## 3. General Principles and Evidence Supporting Immunization during Pregnancy

The unique relationship a mother holds in the protection of the foetus or infant from infection is through the pathway of vertical transmission, whereby maternally derived antibodies cross the placenta to the foetus (IgG) and are transferred through breastfeeding (IgG, IgA, IgM) to the neonates and infants [7]. Before the advent of vaccinations, for the human race to survive, it is plausible to postulate that the maternal exposure to infectious agents offered the sufficient antibodies in the colostrum (breast milk)—the “*first vaccine*” a mother could offer to a newborn infant.

The initial hesitancy in the design and study of vaccine safety and pharmacokinetics in pregnant women has led to a delay in the use of vaccines and their subsequent tailoring within this high-risk group. Importantly, recent research has not demonstrated any adverse maternal or neonatal outcomes associated with the use of vaccinations antenatally [8,9,10,11].

The role of maternal immunisation has recently become a focus for public health campaigns, not only for the prevention of maternal antenatal and post-partum infections but also for the intent to reduce neonatal and early childhood infections through maximising this unique relationship. IgG is the only antibody that is actively transferred across the placenta from the 13th week of pregnancy and this transfer significantly increases during the third trimester. The concentration of fetal IgG in the late second and early third trimester is 25–50% lower than in term infants and there is a debate as to what the optimal timing of the vaccination during pregnancy is [12]. Several clinical trials have identified significantly higher vaccine specific secretory IgA (sIgA) in the breast milk of women vaccinated in the third trimester, with levels remaining elevated up to seven months postpartum [13]. However, further studies are required to demonstrate the impact vaccine-specific secretory IgA has on the incidence or severity of illnesses [9]. Maternal vaccinations have also been suggested to offer protection against non-infectious illnesses during infancy and childhood [14].

## 4. The Current Recommendations for Immunization during Pregnancy and the Evidence

The Centre for Disease Control and Prevention (CDC) [15] recommendations have undergone vast changes and their current vaccination recommendations around pregnancy are contained in Table 1.

Recent studies have demonstrated the benefits of the use of maternal vaccinations in reducing illnesses for young infants, in whom immunization has not yet commenced or been completed [16,17]. The maternal tetanus vaccination has, together with the developments in aseptic techniques and the suitable choice of equipment, resulted in the near eradication of neonatal tetanus [18]. In 2011, Eick et al. demonstrated a reduction in the incidence of laboratory-proven influenza and the requirement for hospital admission in a group of Native American Indians in the neonatal period, with the benefits extending to six months of age following antenatal immunisation [16]. This study supports the findings in similar studies regarding influenza immunisation amongst a range of populations [8]. A study in the UK demonstrated the benefits of the pertussis vaccination in reducing neonatal pertussis infections with a vaccination efficacy of 91% when matched with the controls [17]. Given the increasing incidence of pertussis globally, maternal vaccination can be regarded as a safe strategy to prevent pertussis in infants, as they have the highest risk from this illness with a very high morbidity [19]. In 2013, the Advisory Committee on Immunization Practices published its updated recommendation that a dose of tetanus toxoid, reduced diphtheria toxoid, and acellular pertussis (Tdap) should be administered during each pregnancy, irrespective of their prior history of receiving Tdap. The recommended timing for maternal Tdap vaccination is between 27 weeks and 36 weeks of gestation [20]. Given the rapid evolution of data surrounding this topic, the immunization guidelines are likely to change over time and the American College of Obstetricians and Gynecologists continue to issue updates accordingly [20]. Recent evidence suggests that the pertussis vaccinations administered during 19–37 weeks of gestation are associated with significantly increased antibody levels in the blood of both mothers and their newborns at birth, compared to a placebo or lack of vaccination [19]. Meanwhile, there is no evidence of an increased risk of serious complications such as stillbirth or preterm birth related to the administration of the vaccine during pregnancy [19]. The Pneumococcal vaccine has been demonstrated as safe in pregnancy and is recommended for at-risk antenatal populations on national immunisation guidelines. A recent meta-analysis has shown that maternal influenza immunization may reduce severe pneumonia episodes among infants, particularly those who are too young to be vaccinated against Influenza and Streptococcus pneumonia [21]. The routine antenatal vaccination schedules of many developed countries currently offer influenza and pertussis vaccinations [8].

There is extensive data demonstrating the safety of the influenza vaccination during pregnancy [8,10,16,21]. Relatively fewer studies have examined the safety of Tdap. Donegan et al. reported that among more than 20,000 women in the UK who received Tdap during pregnancy, there was no increased risk of stillbirth, premature birth, low birth weight, or preeclampsia-eclampsia associated with the maternal pertussis vaccination [22]. Kharbanda et al. evaluated whether the receipt of Tdap in 123,494 pregnancies was associated with the increased risks of adverse obstetric or birth outcomes. No effect on the SGA or preterm birth was observed, however, a small increase in the possible/probable chorioamnionitis was observed [23]. However, the authors were unsure of the contribution by heterogeneity in the definition and the diagnosis of chorioamnionitis.

Similar to the indirect community benefits (“herd immunity” or “community immunity”) offered by the optimal uptake of vaccination by the general childhood population, the maternal vaccination, if taken up to sufficient levels, could offer similar benefits to the vulnerable perinatal population who, for many reasons, may be unable to be directly immunized. Maternal immunization is only one component of a multitude of strategies that are used to improve perinatal health outcomes. Other important preventive measures include regular antenatal and perinatal care, the prevention of neonatal hypoxic encephalopathy (HIE), and the promotion of breastfeeding through baby-friendly health initiatives (BFHI).

## 5. Immunization during Pregnancy and the Benefits to Premature Infants

A key priority in preventive neonatology is the reduction or elimination of severe infections and the associated morbidity among premature babies. With the increasing survival of infants with extremely low birth weights (ELBW) [24], including those at the margins of viability [25], the timely and early maternal immunization could offer protection to this immunologically compromised population [26,27]. Traditional prospective antenatal interventions such as the optimisation of maternal nutrition, the timing and administration of antenatal steroids, and the administration of peripartum magnesium sulphate and a group B streptococcal (GBS) screening, are all aimed at reducing the morbidity associated with premature births. Going forward, maternal vaccinations should gain momentum and may also be prospectively offered to confer protection to premature neonates as part of routine care. Influenza, as well as H1N1, during pregnancy, have been shown to increase the rate of premature births and children with low birth weight. Well conducted studies of the influenza vaccine during pregnancy have shown a reduction in the rate of prematurity and other adverse health outcomes [8,10]. The optimal care of ELBW infants has a significant health economic impact and cost-effective preventive methods such as maternal immunization could potentially offer significant cost savings.

## 6. Potential New Unconventional Immunization Options during Pregnancy

Recently, the focus has turned toward the development of antenatal vaccinations against other major neonatal pathogens including the respiratory syncytial virus (RSV), group B streptococcus (GBS), cytomegalovirus (CMV), and rotavirus (RV) [10,28,29,30]. RSV causes a significant respiratory disease burden globally, most markedly in young infants in low- and middle-income countries. The vaccination of women during pregnancy is considered to be the most plausible strategy towards providing direct RSV antibody protection to young infants during this period of the greatest vulnerability [29,31].

Group B streptococcus, found in the vagina or lower gastrointestinal tract of about 10–40% of women of reproductive age, is a leading cause of early invasive neonatal bacterial disease, potentially amenable to prevention through maternal immunization during pregnancy. This also poses the question as to what should be the ideal immunization model for the future—should vaccination be targeted at clearing the infection of GBS colonised women during pregnancy or is the objective in vaccine development for targeted prophylactic vaccination to prevent infections prior to the childbearing age? Following a consultation process with global stakeholders, the World Health Organization (WHO) is proposing priority research, development pathways, and preferred product characteristics for GBS vaccines, with the aim of facilitating and accelerating vaccine licensure, together with policy recommendation for wide-scale use and implementation [32].

A vaccine against the congenital human cytomegalovirus (CMV) infection is a major public health priority. Congenital CMV causes substantial long-term morbidity, particularly sensorineural hearing loss (SNHL), in newborns. Although progress toward the development of a vaccine has been limited by an incomplete understanding of the correlates of protective immunity for the foetus, knowledge of some of the key components of the maternal immune response necessary for the prevention of transplacental transmission is accumulating [33].

With studies currently underway on the safety and efficacy of some of these vaccinations [34,35], antenatal immunization and immunoprophylaxis measures that offer neonatal protection against other infectious agents, including Hepatitis E and Zika, are still in the developmental phase [36,37]. A recent US study observed that pregnant women were worried about Zika, yet had significant gaps in their factual knowledge. Most women reported that they would get vaccinated if a vaccine was available [36].

## 7. Immunization during Pregnancy against Non-Infectious Neonatal Morbidities

While the prevention of infectious diseases during infancy and childhood through an antenatal vaccination is still in development, and yielding results, this has also opened up other areas of interest including vaccinations towards other potential childhood illnesses through the induction of antibodies to non-infectious antigens. Animal studies have demonstrated the benefit in reducing atherosclerosis and allergies so far [38,39], with an interest in the development of vaccinations to prevent childhood cancers through antibody formation toward tumour-associated-antigens [14], with maternal immunization as a vehicle for the prevention of non-infectious illnesses during infancy and childhood.

## 8. Immunization during Pregnancy and the Potential Impact on the Infants’ Immune Response

The literature also raises questions on whether maternal immunization poses any potential negative impacts on the early life immunity by means of maternal antibodies interfering with the development of the infant immune responses, though it is unclear if such an interference is clinically significant [2]. Thus, the risk/benefit of maternal immunization for both the mother and the foetus should be carefully weighed. In addition, it is critical to fully understand the mechanisms by which IgG is transferred across the placenta in order to develop optimal maternal and infant immunization strategies [2]. The ability of maternal IgG antibodies to be transported readily across the healthy intact placenta depends on many different factors including the gestational age in the pregnancy, the nature and timing of the immunization, and the presence of maternal HIV or malaria infections [4]. Although much evidence has highlighted the benefits of maternal immunization, some studies have also shown a potential interference between maternally derived IgG antibodies and infant antibody responses [40]. Specifically, maternally derived IgG antibodies can inhibit immune responses against antigens after the primary vaccination in early infancy, a phenomenon termed “immunological blunting”. This blunting usually dissipates after the booster dose [41].

The long-term adverse effects of maternal immunization on infants have not been reported. However, the continued monitoring of the safety of various vaccines administered in a geographically diverse population during pregnancy will be important to gain and galvanize professional and public confidence.

## 9. Immunization during Pregnancy and the Impact on the Perinatal Antimicrobial Resistance

A critical concern for public health systems worldwide is the emergence of antimicrobial resistance. Maternal immunization as part of a life-course immunization approach could potentially offer a significant contribution by reducing the maternal, perinatal, and neonatal antibiotic exposure, which has been increasing in both developed and resource-limited settings [42]. Vaccines have been proposed as an essential intervention to alleviate the pressures on growing antibiotic resistance, complementing improvements in diagnostic testing, antibiotic stewardship, and drug pipelines. Immunization as a measure to reduce antimicrobial resistance is gaining momentum among public health professionals and policymakers. However, the awareness among obstetric, midwifery, and neonatal communities would benefit from further education on vaccines. The decision to introduce or amend vaccination programmes is routinely based on mathematical modelling [43]; however, few mathematical models address the impact of vaccinations on antibiotic resistance and nil, specifically through immunization during pregnancy.

## 10. Existing and Emerging Challenges for Maternal Immunization and the Role of Health Providers

**Figure 1:**
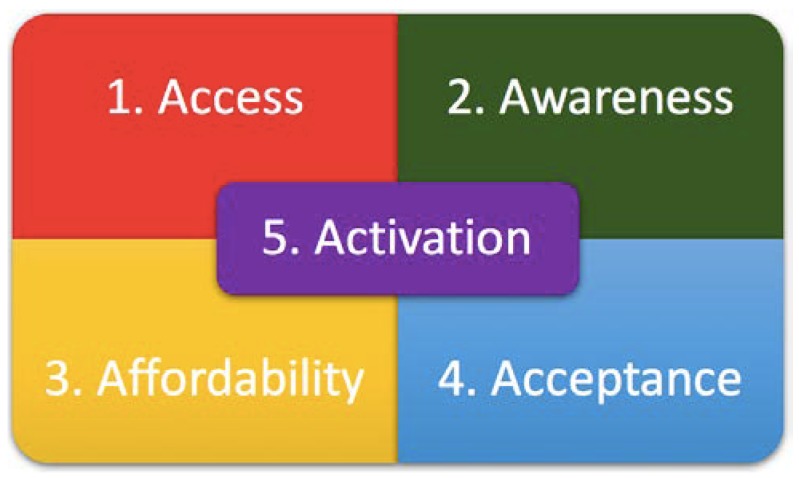
The key alignments for successful vaccination programmes.

Vaccination programmes globally are facing new challenges, including vaccine hesitancy, anti-vaccine lobbying, and the emergence of new communicable diseases. Globally, the success of vaccination programmes, in general, is achieved with the alignment of the “five As”—Access, Affordability, Awareness, Acceptance, and Activation (Figure 1). Maternal immunization programmes are no exception to this ground rule.

Successful maternal vaccination programmes could be materialized through the alignment of the “five As”—Access, Affordability, Awareness, Acceptance, and Activation.

**Access:** Access is the availability of the recommended vaccines during routine antenatal care. That is, that the vaccine is widely available and accessible to all. The means of producing, storing, and transporting the vaccine should be available, including the maintenance of cold chains in resource-limited settings. Maternal immunization programmes should be properly funded so that healthcare workers can get involved without detriment to other practice responsibilities.

**Affordability:** Affordability refers to the financial and non-financial burdens associated with vaccine uptake. A lower cost at the point of delivery should be the aim so that maximum uptake can be ensured. This is of particular significance to maternal immunization in resource-limited settings, such as in Africa.

**Awareness:** Awareness is the maternal knowledge and information provided regarding the availability, the recommendations, as well as the risks and benefits. Most successful vaccination initiatives are often built on pre-existing healthcare-related and community-rooted relationships. The planning and supervision of adequate staff training is crucial and the public confidence in the safety of the vaccines is critical to the success of maternal immunization programmes.

**Acceptance:** Acceptance relates to the individual’s risk-benefit values and the impacts on the individual uptake of the offered vaccination. Understanding the effect of the social determinants on immunization programmes in various countries is important in order to address them with the purpose of optimizing vaccination. Trust is considered an important factor in enhancing the acceptance of maternal vaccinations and developing effective communication.

**Activation:** Activation refers to the proactive steps taken by healthcare providers and public health authorities to encourage vaccination through education, information dissemination, and reminders. An active anti-vaccine lobby thrives today in spite of the undeniable success of vaccination programmes. False alarms and misguided safety concerns in some countries have led to a fall in the coverage of vaccinations. The activation and optimisation of managing risk communication is also paramount. 

A critical factor that is shaping public attitudes towards vaccination is the interaction with health professionals. Poor communication can contribute to the rejection of vaccinations or a dissatisfaction with the care. The promotion of maternal vaccination uptake may be augmented with a framework for health professionals for effective and respectful communication. Educational material targeting pregnant women and the professional education and support of antenatal health care providers are needed in order to increase the awareness and recommendation of vaccinations [44]. Studies have shown that many pregnant women look to their obstetricians to guide their prenatal and antenatal care. A strong provider recommendation remains the greatest impetus to increase vaccine uptake [45]. The strong recommendation of providers and the availability of maternal vaccines in OB/GYN offices are keys to improving vaccine uptake. Attention must be paid to the further development of intervention techniques that address unique barriers such as vaccine cost, storage concerns, and misinformation about vaccine safety [45]. Both healthcare providers and expectant mothers should continue to be educated on the importance and safety of the appropriate immunizations during pregnancy. The extended role of Obstetrician-Gynaecologists as vaccinators and the developing systems approaches to facilitating vaccinations for women both during and outside of their pregnancies has been suggested [46,47]. However, financial barriers and the infrequent use of evidence-based strategies for increasing vaccination uptake may be hindering the delivery of a broader complement of adult vaccines in the offices of obstetricians/gynaecologists [48]. 

## 11. The Impact of Public Perception, Social Media, and Modern Communications on Maternal Immunization

In light of the growing dependence on social media for evidence and healthcare-related decision making, it is important to disseminate the appropriate messages from the very outset to both professionals and the intended population using modern communication channels. It is also important that the information conveyed is with the best evidence, honest, and transparent [49]. This would be paramount in building trust in maternal immunization, especially at a time when public perception and the resultant health behaviour could easily be derailed by misinformation. Highly publicised vaccine scares and recent debates about the risks of vaccinations suggest that the public trust in immunisation programmes is fragile.

## 12. The Role of Regulation, Safety Assessments, and Appropriate Risk Communications on Maternal Immunization

**Figure 2:**
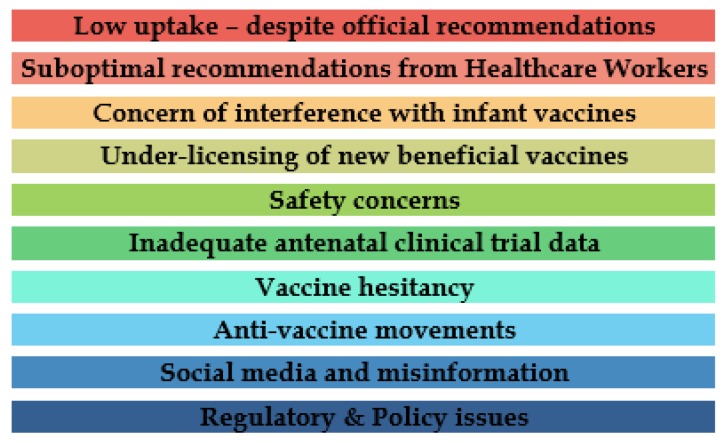
The barriers to maternal vaccinations.

Appropriate legislative and regulatory changes, as well as recommendations from national professional bodies, could address the key barriers to advancing further research in the field of maternal immunization [50]. Five procedural principles (another set of “five As”) regarding public risk communication would be worth considering when highlighting the issues around maternal vaccination—Assembling the evidence, Acknowledgement of public perspectives, Analysis of options, Authority in charge, and Audience interaction [51]. Figure 2 summarises the main barriers to maternal immunization.

Although the CDC recommends the use of vaccines during pregnancy, certain ethical, policy, educational, and research barriers need to be addressed in order to improve the uptake of the currently recommended vaccines and to promote the development of additional maternal immunizations [52]. The barriers also include doubt about the effectiveness of the vaccine, lack of knowledge about the burden of the disease, complacency, and not feeling oneself at risk from the infection. The National Vaccine Advisory Committee (NVAC) established the Maternal Immunization Working Group in 2012 to conduct these assessments and to provide recommendations for overcoming the identified barriers. These are summarised below:

(1) Collaboration with bioethics experts, regulatory agencies, and the scientific community to optimize the design of studies in order to minimize the risk of interventions in the research of pregnancy; (2) the relevant regulations and policies should be modified to indicate that pregnant women are not a vulnerable population for the purposes of ethical reviews; (3) although there is a concern that including pregnant women in a study of new vaccines could lead to foetal harm, it is important to recognize that excluding pregnant women from research could also lead to harm; (4) another challenge that contributes to the exclusionary climate toward pregnant women in clinical trials is that currently, researchers must justify the inclusion of pregnant women to regulatory authorities and specify the special protections that will be in place during product testing. Interestingly, there is no requirement to justify the exclusion of pregnant women from a protocol; (5) a more thorough understanding of the vaccine-preventable disease burden among infants in the first 6 months of life would also help to accurately determine the effectiveness of maternal immunizations on both the infant and the mother and could help justify the importance of this intervention to policy makers and to the general public; (6) well-established post-marketing reporting and surveillance systems allow for the study of the adverse events of vaccines currently in use and for the research of diverse safety outcomes, even in the absence of reports of a specific adverse event; (7) professional societies and maternal immunization stakeholders have a critical role in educating providers about the benefits of involving pregnant women in clinical trials. Their community engagement efforts are essential in order to support a shift towards including pregnant women; (8) obstetric providers are the most trusted advisors of a pregnant patient and are uniquely positioned to advocate for the increased uptake of vaccinations during pregnancies and for the enhanced participation of pregnant women in clinical research.

The collaboration of bioethics experts, regulatory agencies, and the scientific community to optimise the design of studies in order to minimise the risk of interventions in the research of pregnancy, including trials of vaccines, would be important [50]. Recently, the Global Alignment of Immunization safety assessment in the Pregnancy Project (http://gaia-consortium.net) developed a set of guidelines for the collection of essential safety data in clinical trials involving the administration of vaccines to pregnant women [53]. Both scientific and regulatory considerations remain challenging against the licensure of vaccines, specifically for maternal immunization [4].

General socio-economic factors influencing the universal uptake of vaccination in a given population is reflected on maternal immunization uptake as well. Perhaps this could be exaggerated for vaccination during pregnancy in the resource-limited settings. Major socio-demographic factors limiting maternal immunization noted in a recent research study from Africa highlighted the inadequate financial and human resources which translated to the inadequate delivery and logistics management of vaccination services [54]. Health care providers are limited by the poor attendance of antenatal care and the inadequate knowledge of vaccinating pregnant women. The barriers to the patients are due to lack of education and knowledge on immunization during pregnancy and socioeconomic factors such as low income and high parity [54].

## 13. Conclusions

A paradigm shift in maternal vaccination to “optimize the evidence, reduce the risk, communicate the awareness, and encourage the uptake” would have a significant positive contribution to preventive neonatology. Maternal vaccinations merit appropriate priority in a life-course immunization approach and it is perhaps the only immunization whereby two generations benefit directly from a single input. There is considerable evidence supporting the safety, efficacy, immunogenicity, and effectiveness of immunization during pregnancy. Generating more evidence on the use and timing of maternal immunization, as well as identifying and addressing the barriers preventing a wider vaccination uptake, would be a few of the key challenges to the vaccine community. Further research should fill in the knowledge gaps and strengthen the evidence for the efficacy and safety of maternal immunization. Given the ongoing research into the use of antenatal vaccinations and, particularly, its use to prevent foetal and neonatal morbidity, the lifelong immunization strategy is a key component that may be more fully explored in the coming years. A roadmap for the interdisciplinary collaborations to integrate maternal immunization with improving access to preventive and other healthcare interventions for all the women throughout pregnancy would pave the way for preventive neonatology.

## Figures and Tables

**Table 1:** The summary table of the vaccination in pregnancy Centre for Disease Control and Prevention (CDC) Recommendations.

Population	Vaccine	Type of Vaccine
**All Pregnant Women**	Influenza	Inactivated
	Tetanus Diphtheria and acellular Pertussis	Toxoid, Inactivated
**Pregnant Women at Risk**	Hepatitis A	Inactivated
	Hepatitis B	Inactivated
	Meningococcal	Inactivated
	Pneumococcal	Inactivated
	Human Papilloma Virus	Inactivated
	Tetanus and Diphtheria	Toxoid
Postpartum *	Measles Mumps and Rubella	Live
	Varicella	Live

* If a woman is seronegative for rubella and varicella antibodies, where possible, an MMRV (Measles Mumps Rubella Varicella) vaccine course is recommended prior to pregnancy to protect the unborn child.

## References

[1] Watson R.S., Carcillo J.A., Linde-Zwirble W.T., Clermont G., Lidicker J., Angus D.C. (2003). The Epidemiology of Severe Sepsis in Children in the United States. Am. J. Respir. Crit. Care.

[2] Fouda G.G., Martinez D.R., Swamy G.K., Permar S.R. (2018). The Impact of IgG Transplacental Transfer on Early Life Immunity. Immunohorizons.

[3] Bonanni P., Chiamenti G., Conforti G., Maio T., Odone A., Russo R., Scotti S., Signorelli C., Villani A. (2017). The 2016 Lifetime Immunisation Schedule, approved by the Italian scientific societies: A new paradigm to promote vaccination at all ages. Hum. Vaccines Immunother..

[4] Kachikis A., Englund J.A. (2016). Maternal immunisation: Optimising protection for the mother and infant. J. Infect..

[5] Lindsey B., Kampmann B., Jones C.E. (2013). Maternal immunization as a strategy to decrease susceptibility to infection in newborn infants. Curr. Opin. Infect. Dis..

[6] Perrett K.P., Nolan T.M. (2017). Immunisation During Pregnancy: Impact on the Infant. Pediatr. Drugs.

[7] Faucette A.N., Unger B.L., Gonik B., Chen K. (2015). Maternal vaccination: Moving the science forward. Hum. Reprod. Update.

[8] Salam R.A., Das J.K., Soeandy C.D., Bhutta Z. (2015). Impact of Haemophilus influenzae type B (Hib) and viral influenza vaccinations in pregnancy for improving maternal, neonatal and infant health outcomes. Cochrane DB Syst. Rev..

[9] Chaithongwongwatthana S., Yamasmit W., Limpongsanurak S., Lumbiganon P., Tolosa J.E. (2015). Pneumococcal vaccination during pregnancy for preventing infant infection. Cochrane DB Syst. Rev..

[10] McMillan M., Porritt K., Kralik D., Marshall H.S. (2015). Influenza vaccination during pregnancy: A systematic review of fetal death, spontaneous abortion, and congenital malformation safety outcomes. Vaccine.

[11] Bonde U., Joergensen J.S., Lamont R.F., Mogensen O. (2016). Is HPV vaccination in pregnancy safe?. Hum. Vaccines Immunother..

[12] Calvert A., Jones C.E. (2017). Placental transfer of antibody and its relationship to vaccination in pregnancy. Curr. Opin. Infect. Dis..

[13] Jones C.E., Calvert A., le Doare K. (2018). Vaccination in Pregnancy—Recent Developments. Pediatr. Infect. Dis. J..

[14] Riccardo F., Real A., Voena C., Barutello G. (2017). Maternal Immunization: New Perspectives on Its Application Against Non-Infectious Related Diseases in Newborns. Vaccines.

[15] The Centre for Disease Control and Prevention, Pregnancy and Vaccination (2016). Vaccines for Pregnant Women. https://www.cdc.gov/vaccines/pregnancy/pregnant-women/index.html.

[16] Eick A.A., Uyeki T.M., Klimov A., Hall H., Reid R., Santosham M., O’Brien K.L. (2011). Maternal Influenza Vaccination and Effect on Influenza Virus Infection in Young Infants. Arch. Pediatr. Adolesc. Med..

[17] Dabrera G., Amirthalingam G., Andrews N., Campbell H., Ribeiro S., Kara E., Fry N.K., Ramsay M. (2014). A Case-Control Study to Estimate the Effectiveness of Maternal Pertussis Vaccination in Protecting Newborn Infants in England and Wales, 2012–2013. Clin. Infect. Dis..

[18] Khan R., Vandelaer J., Yakubu A., Raza A.A., Zulu F. (2015). Maternal and neonatal tetanus elimination: From protecting women and newborns to protecting all. Int. J. Womens Health.

[19] Furuta M., Sin J., Ng E.S., Wang K. (2017). Efficacy and safety of pertussis vaccination for pregnant women—A systematic review of randomised controlled trials and observational studies. BMC Pregnancy Childbirth.

[20] Committee on Obstetric Practice, Immunization and Emerging Infections Expert Work Group (2017). Committee Opinion No. 718: Update on Immunization and Pregnancy: Tetanus, Diphtheria, and Pertussis Vaccination. Obstet. Gynecol..

[21] Omer S.B., Clark D.R., Aqil A.R., Tapia M.D., Nunes M.C., Kozuki N., Steinhoff M.C., Madhi S.A., Wairagkar N. (2018). Maternal Influenza Immunization and Prevention of Severe Clinical Pneumonia in Young Infants: Analysis of Randomized Controlled Trials Conducted in Nepal, Mali, and South Africa. Pediatr. Infect. Dis. J..

[22] Donegan K., King B., Bryan P. (2014). Safety of pertussis vaccination in pregnant women in UK: Observational study. BMJ.

[23] Kharbanda E.O., Vazquez-Benitez G., Heather S., Lipkind H.S., Klein N.P., Cheetham T.C., Naleway A., Omer S.B., Hambidge S.J., Lee G.M. (2014). Evaluation of the Association of Maternal Pertussis Vaccination With Obstetric Events and Birth Outcomes. JAMA.

[24] Shim J.W., Jin H.-S., Bae C.-W. (2015). Changes in Survival Rate for Very-Low-Birth-Weight Infants in Korea: Comparison with Other Countries. J. Korean Med. Sci..

[25] Ancel P.-Y., Goffinet F. (2015). EPIPAGE-2 Writing Group. Survival and Morbidity of Preterm Children Born at 22 through 34 Weeks’ Gestation in France in 2011Results of the EPIPAGE-2 Cohort Study. JAMA Pediatr..

[26] Philip R.K. (2018). Margins of viability. Ir. J. Med. Sci..

[27] Shah P.S., Lui K., Sjors G., Mirea L., Reichman B., Adams M., Modi N., Darlow B.A., Kusuda S., Feliciano L.S. (2016). Neonatal Outcomes of Very Low Birth Weight and Very Preterm Neonates: An International Comparison. J. Pediatr..

[28] Khan A.I., Ali M., Chowdhury F., Saha A., Khan I.A., Khan M.A., Akter A., Asaduzzaman M., Islam M.T., Kabir A. (2017). Safety of the oral cholera vaccine in pregnancy: Retrospective findings from a subgroup following mass vaccination campaign in Dhaka, Bangladesh. Vaccine.

[29] Scheller N.M., Pasternak B., Molgaard-Neilsen D., Svanstrom H., Hviid A. (2017). Quadrivalent HPV Vaccination and the Risk of Adverse Pregnancy Outcomes. New Engl. J. Med..

[30] Sukumaran L., McCarthy N.L., Kharbanda E.O., Weintraub E., Vazquez-Benitez G., McNeil M.M., Li R., Klein N., Hambridge S.J., Naleway A.L. (2015). Safety of Tetanus, Diphtheria, and Acellular Pertussis and Influenza Vaccinations in Pregnancy. Obstet. Gynecol..

[31] Munoz F.M. (2015). Respiratory syncytial virus in infants: Is maternal vaccination a realistic strategy?. Curr. Opin. Infect. Dis..

[32] Vekemans J., Moorthy V.S., Friede M., Alderson M.R., Meulen A.S.-T., Baker C.J., Heath P.T., Madhi S.A., Doare K.M., Saha S.K. (2018). Maternal immunization against Group B streptococcus: World Health Organization research and development technological roadmap and preferred product characteristics. Vaccine.

[33] Schleiss M.R., Permar S.R., Plotkin S.A. (2017). Progress toward Development of a Vaccine against Congenital Cytomegalovirus Infection. Clin. Vaccine Immunol..

[34] Nuccitelli A., Rinaudo C.D., Maione D. (2015). Group B Streptococcus vaccine: State of the art. Ther. Adv. Vaccines.

[35] Neuzil K.M. (2016). Progress toward a Respiratory Syncytial Virus Vaccine. Clin. Vaccine Immunol..

[36] Fraiz L.D., de Roche A., Mauro C., Catallozzi M., Zimet G., Shapiro G., Rosenthal S.L.U.S. (2017). pregnant women’s knowledge and attitudes about behavioral strategies and vaccines to prevent Zika acquisition. Vaccine.

[37] Omer S.B. (2017). Maternal Immunisation. N. Engl. J. Med..

[38] Yamashita T., Freigang S., Eberle C., Pattison J., Gupta S., Napoli C., Palinski W. (2006). Maternal Immunization Programs Postnatal Immune Responses and Reduces Atherosclerosis in Offspring. Circ. Res..

[39] Melkild I., Groeng E.C., Leikvold R.B., Granum B., Lovik M. (2002). Maternal allergen immunization during pregnancy in a mouse model reduces adult allergy-related antibody responses in the offspring. Clin. Exp. Allergy.

[40] Niewiesk S. (2014). Maternal antibodies: Clinical significance, mechanism of interference with immune responses, and possible vaccination strategies. Front. Immunol..

[41] Edwards KM. (2015). Maternal antibodies and infant immune responses to vaccines. Vaccine.

[42] Ginsburg A.S., Klugman K.P. (2017). Vaccination to reduce antimicrobial resistance. Lancet Glob. Health.

[43] Atkins K.E., Lafferty E.I., Deeny S.R., Davies N.G., Robotham J.V., Jit M. (2017). Use of mathematical modelling to assess the impact of vaccines on antibiotic resistance. Lancet Infect. Dis..

[44] Wiley K., Massey P., Cooper S., Wood N., Ho J., Quinn H.E., Leask J. (2013). Uptake of influenza vaccine by pregnant women: A cross-sectional survey. Med. J. Aust..

[45] Bethancourt C.-N., Wang T.L., Bocchini J.A. (2017). Vaccination during pregnancy: First line of defense for expecting mothers and vulnerable young infants. Curr. Opin. Pediatr..

[46] Swamy G.K., Heine R.P. (2015). Vaccinations for Pregnant Women. Obstet. Gynecol..

[47] Bradley S.L., Ehrenthal D.B. (2014). Commentary on Maternal immunization: Clinical experiences, challenges, and opportunities in vaccine acceptance. Hum. Vaccines Immunother..

[48] O'Leary S.T., Riley L.E., Lindley M.C., Aliison M.A., Crane L., Hurley L., Beaty B.L., Brtnikova M., Collins M., Albert A.P. (2017). Immunization Practices of U.S. Obstetrician/Gynecologists for Pregnant Patients. Am. J. Prev. Med..

[49] Philip R.K., Shapiro M., Peterson P., Glismann S., van Damme P. (2016). Is It Time for Vaccination to Go Viral?. Pediatr. Infect. Dis. J..

[50] Beigi R.H., Omer S.B., Thompson K.M. (2018). Key steps forward for maternal immunization: Policy making in action. Vaccine.

[51] Bouder F. (2015). Risk Communication of Vaccines: Challenges in the Post-Trust Environment. Curr. Drug Saf..

[52] Overcoming Barriers and Identifying Opportunities for Developing Maternal Immunizations: Recommendations from the National Vaccine Advisory Committee Approved by the National Vaccine Advisory Committee. http://journals.sagepub.com/doi/abs/10.1177/0033354917698118.

[53] Bonhoeffer J., Kochhar S., Hirschfeld S., Heath P.T., Jones C.E., Bauwens J., Honrado A., Heininger U., Munoz F.M., Eckert L.O. (2016). Global alignment of immunization safety assessment in pregnancy—The GAIA project. Vaccine.

[54] Pathirana J., Nkambule J., Black S. (2015). Determinants of maternal immunization in developing countries. Vaccine.

